# Nutritional value of suckler beef from temperate pasture systems

**DOI:** 10.1016/j.animal.2021.100257

**Published:** 2021-07

**Authors:** M.R.F. Lee, G.A. McAuliffe, J.K.S. Tweed, B.A. Griffith, S.A. Morgan, M.J. Rivero, P. Harris, T. Takahashi, L. Cardenas

**Affiliations:** a Rothamsted Research, North Wyke, Okehampton, Devon EX20 2SB, UK; b University of Bristol, Bristol Veterinary School, Langford, Somerset S40 5DU, UK; c Aberystwyth University, Institute of Biological, Environmental and Rural Science, Gogerddan, Aberystwyth, Ceredigion SY23 2EB, UK

**Keywords:** Fatty acids, Grassland, Nutritional quality, Red meat, Sustainability assessment

## Abstract

The role of beef in human diets has been questioned over the last few decades, due largely to its typically high mass-based carbon footprint. However, recent advancements in sustainability literature challenge this paradigm based on the new theory that climate impacts of food commodities should be measured relative to their overall nutritional value rather than their nominal mass. This shift has opened a new opportunity for the global beef industry, and especially for pasture-based systems that can avoid food-feed competition for land and other resources, as beef is a nutritionally dense food. Nonetheless, the sector’s true capability to supply a wide range of nutrients for humans, consistently across multiple systems under multiple weather patterns, has not been well-documented. Using whole-system datasets from the North Wyke Farm Platform in the South West of England, we investigated the nutritional value of beef produced from the three most common pasture systems in temperate regions: permanent pasture (**PP**), grass and white clover (**GWC**) and a short-term monoculture grass ley (**MG**). Beef produced from these three pasture systems was analysed for key nutrients (fatty acids, minerals and vitamin E) over three production cycles (2015–2017) to determine potential differences between systems. Fatty acid, mineral and vitamin E profiles of the pasture and silage fed to each group were also assessed, with subtle differences between pastures reported. For beef, subtle differences were also observed between systems, with GWC having higher omega-6 polyunsaturated fatty acid (**PUFA**) concentrations than PP and MG. However, the overall nutritional quality of beef was found to be largely comparable across all systems, suggesting that temperate pasture-based beef can be classified as a single commodity in future sustainability assessments, regardless of specific sward types. A 100 g serving of temperate pasture-based beef was found to be a high source (>20% recommended daily intake: **RDI**) of protein, monounsaturated fatty acids, saturated fatty acids, vitamins – B2, B3, B12 and minerals – Fe, P, Zn; a good source (10–19% RDI) of vitamin – B6 and mineral – K; and a complementary source (5–9% RDI) of omega-3 PUFA, vitamin – B9 and minerals – Cu, Mg, Se. The nutritional value of a food item should be used in defining its environmental cost (e.g. carbon footprint) to make fair comparisons across different food groups (e.g. protein sources). Here, we showed that pasture-based beef had a nutrient indexed carbon footprint of between 0.19 and 0.23 Kg CO2-eq/1% RDI of key nutrients.

## Implications

Accurate quantification of the nutritional value of a food item is a prerequisite for any sustainability assessment of agricultural systems. Within the context of pasture-based beef production under temperate climates, this study holistically evaluates the systems’ capabilities to supply both macronutrients and micronutrients.

## Introduction

Grasslands in their widest sense are the largest terrestrial ecosystem, covering 52.5 million km^2^, which is equivalent to 40.5% of the world’s ice-free landmass (FAOSTAT, 2020). Whilst these grasslands are diverse (including the Steppes of Asia, Campos and Pampas of South America, Prairie grasslands of North America, Dry and Rangelands of South West and North Australia, Savannas of Africa and the temperate grasslands of Europe and New Zealand), they all support ruminant livestock production as a food source, to some level. This is especially true for beef production, and in temperate climates, most beef enterprises seek the best use of home-grown pasture with a combination of grazing and conserved forage feeding, the latter predominately when conditions are unsuitable for grazing or the herbage available is not sufficient to meet animals’ nutritional requirements (Moloney et al., 2018). Although cereal-based feeds can have vital roles in complementing grazed pasture and conserved forages, such as increasing total diet DM intake and rectifying nutritional imbalances (especially for high-yielding dairy cows), grazed pasture and forage are the single most important feed for ruminants (Wilkinson and Lee, 2018). Pastures not only provide the lowest unit-cost of production, but also exemplify the positioning of ruminants in terms of sustainability as they utilize resources which could not otherwise be used directly for human nutrition and at the same time return carbon to soil through their manures (Wilkinson et al., 2020, Eisler et al., 2014).

This sustainable positioning of ruminants is critically important in the face of climate change (Rivero et al., 2021) as the sector is typically associated with high greenhouse gas emissions per unit of product (Poore and Nemecek, 2018). Consequently, levels and patterns of beef consumption and production must align with environmental targets while also considering social (e.g. animal welfare) and economic (e.g. rural communities) security. This ‘sustainable’ approach may therefore require strict adherence to principles such as employing livestock as utilizers of industrial waste-streams and maintaining animals only on marginal lands not suitable for arable cultivation, e.g. grasslands on hilly or otherwise difficult-to-manage terrains (Eisler et al., 2014, Van Zanten et al., 2018, Gill et al., 2018).

The role livestock products play as part of a healthy diet has also been widely debated, predominately due to an association of red and processed meat with colorectal cancer (Bellavia et al., 2016) and an on-going concern regarding saturated fats and their links to cardiovascular disease (Wilkins et al., 2017). However, the evidence suggests that, although processed meats are indeed detrimental to human health, particularly when consumed in large quantities, lean red meat poses a negligible risk to health (Micha et al., 2010) when consumption levels are within dietary guidelines (NHS, 2019). When consumed within these guidelines, the nutrient concentration of livestock products, due to their high provision of vitamins, minerals and essential amino and fatty acids, often has a positive impact on human health (Givens, 2018). Furthermore, when the effect of over-consumption is statistically eliminated from an omnivorous diet, the environmental impact of ruminant livestock products is comparable to mono-gastric livestock (McAuliffe et al., 2018a) and in health terms little different to solely plant-based diets (Asvatourian et al., 2018).

The impact of production systems on the nutritional value of ruminant products, including meat, has been well researched, especially between pasture and cereal-based systems (Nuernberg et al., 2005, Warren et al., 2008a, Warren et al., 2008b). Studies have shown higher levels of beneficial fatty acids such as long chain omega-3 and conjugated linoleic acid and certain vitamins (E and Carotene (pro-vitamin A)) on high forage diets compared to more cereal-based diets (as reviewed by Daley et al., 2010). However, few studies have compared differences *within* pasture-based production systems where different combinations of plant species and management strategies may influence meat quality not only in terms of fatty acid composition and content but also vitamins and minerals. This gap in the literature is unsatisfactory given the greater role pastures will likely play in the future beef industry to avoid food-feed competitions for land resources.

Motivated by this observation, this study investigated the nutritional value of beef from the three most common pasture systems in temperate regions: permanent pasture (grasslands which have not been reseeded for 5 years or more), grass and white clover (*Trifolium repens* L.) leys (driven by legume N-fixation with reduced inorganic fertilizer inputs) and monoculture grasslands (short-term leys sown to optimize new germplasm and/or used in rotations).

## Material and methods

### Experimental design and grassland systems

The experiment was conducted between 2015 and 2017 on the North Wyke Farm Platform (**NWFP**) ca. 63 ha of Rothamsted Research’s North Wyke farm in South West England (50°46′10″N, 3°54′05″W) established as a UK National Capability to investigate the sustainability of temperate grazing livestock systems. The NWFP consists of three self-contained suckler finisher beef and sheep farmlets (small farms; ca. 21 ha) managed as different sward types. The establishment of the facility and the level of instrumentation is described in detail by Orr et al. (2016) and an assessment of sustainability indicators is outlined in Takahashi et al. (2018).

The three NWFP grassland systems were based on: (i) Permanent pasture (**PP**); no change from the baseline assessment (botanical composition and more detail regarding the swards can be found in Orr et al., 2016, Orr et al., 2019); (ii) Perennial ryegrass (*Lolium perenne* L.) and white clover – (**GWC**) with a 70:30 land cover target, respectively, representing a reduced reliance on artificial nitrogen (**N**) fertilizer through legume biological N-fixation; iii) Monoculture Grass (perennial ryegrass) Monoculture – (**MG**) representing a reseeded short-term ley. The grass and white clover choice of cultivars (cv. AberMagic and AberHerald, respectively) was based on the UK’s recommended list of latest germplasm (BGS, 2013). Following sward establishment, the PP and MG pastures received standard N, phosphorus (**P**) and potassium (**K**) fertilization, whereas the GWC fields received a lower amount of N, predominately in the form of farmyard manure produced during winter housing under each system, as described by Orr et al. (2019).

Calves were sourced at weaning from the main North Wyke farm spring-calving Hereford-Friesian suckler herd sired by a Charolais bull. After allocation (*n* = 30; balanced across treatment for steers and heifers each year) to each pasture system (PP, GWC or MG), the calves were housed, with each farmlet having separate housing, typically from October to April (depending on annual meteorological conditions; Supplementary Material S1) to avoid destruction of soil structure during the wet season. At turnout, cattle went to their respective farmlet pastures and were continuously stocked as described by Orr et al. (2019) until they reached a target fat score of ‘4L’ with predicted weights of 580 kg for heifers and 650 kg for steers. During the housing period, animals were offered *ad libitum* silage harvested from their own allocated systems mixed with general purpose minerals (FeedCo Ltd., Exeter, UK) in a forage wagon to provide ca. 80 g/head/d. The minerals were formulated to contain the following values per kg of dry product: Vitamins (International Units): A 400 000; D3 80 000 and E 1 500. Minerals: calcium (**Ca**) iodate 400 mg; cobalt (Co) (III) carbonate 90 mg; copper (Cu) (II) sulphate pentahydrate 1.13 g; dicopper chloride trihydroxide 188 mg; manganese (Mn) (II) oxide 3 000 mg; zinc (Zn) oxide 4 000 mg and sodium (Na) selenite 35 mg. While the NWFP’s general principle is to finish cattle solely off pasture and silage, depending on the quantity and quality of silage produced each year, strategic supplementary feed may be required. This is predominately delivered through a Greenfeed© system (C-lock, Rapid City, South Dakota, USA) installed in each housing unit to assess methane emissions from enteric fermentation (as part of the wider NWFP instrumentation), which provides on average 1 kg/head/d of pelleted sugar-beet pulp (*Beta vulgaris*) or grass pellets. During the reporting periods, all animals during housing received 1 kg/head/d of grass pellets in 2015 and 1 kg/head/d of sugar-beet pulp in 2016–2017, as a means of encouraging use of the Greenfeed© system (data not shown). Cattle are housed in barns deep-bedded with barley (*Hordeum vulgare*) straw, and farm yard manure produced is stored temporarily in middens until pastures are ready to receive it after the first silage cut in spring.

### Sample collection methods

Data for the present study were collected across 3 years between 2015 and 2017. Samples of silage as fed during housing were collected once a week, frozen and subsequently bulked across a month (−20 °C) prior to analysis. Pasture samples were collected during grazing from fields occupied by cattle once a fortnight or on movement to a new field. Samples were subsequently frozen, bulked across a month and stored (−20 °C) until analysis. Pasture sampling was performed in a W-shaped transect across the grazed fields with samples cut 5 cm above soil level. Animals were weighed and condition scored every 2 weeks. The first five Hereford-Friesian × Charolais steers to finish from each pasture system within each year were submitted for full meat quality evaluation. The decision to limit the sample to early steer finishers was taken to minimize the impact of inter-animal heterogeneity on meat quality that is unattributable to the treatment (e.g. the portion of average daily gains explained by individual animal genetics and sex rather than pasture systems), as the maximum overall sample size logistically feasible (i.e., 15 animals per year with 5 animals per farmlet) did not allow statistically meaningful random sampling across all Hereford-Friesian × Charolais steers and heifers. Postslaughter hot carcass weights were recorded excluding kidney knob and channel fat. External fatness was assessed using the European Commission carcass classification scheme as described by Kempster et al. (1986) before transfer to a chiller at 2 °C.

### Meat preparation and quality assessment

At 48 h postslaughter, a 250 mm-long section of the hind loin joint containing the *M. longissimus lumborum* muscle was removed from the left side of each carcass posterior to the 10th rib and deboned. A 20 mm-thick steak was cut and dissected to remove subcutaneous adipose tissue, vacuum-packed and frozen at −20 °C for subsequent analysis of vitamin E, fatty acids and minerals (see below). Another section of the loin was vacuum-packed and conditioned at 1 °C for a further 8 days. After this, four 20 mm-thick steaks were cut and packed individually in modified atmosphere packs (O_2_:CO_2_: 75:25) and subjected to simulated retail display (4 °C, 700 lux for 16 h). Colour (L*a*b*) coordinates (CIE, 1986) were measured daily on the surface of the steaks through the film using a Minolta Chromameter CR200 (Minolta Camera Company, Milton Keynes, UK) to quantify the full retail display life of the steaks. The chromameter was standardized against a white tile (L* = 97.78, a* = 0.19, b* = 1.84) covered in the modified atmosphere pack top web film and checked against a red plate (L* = 23.0, a* = 24.3, b* = 11.5) also covered in the modified atmosphere pack top web film. Colour saturation (chroma), which is a measure of the intensity of the red colour, was calculated from the formula [(a*)^2^ + (b*)^2^]^0.5^, with loss of shelf life determined when the chroma dropped below a threshold of 18.

The remaining section of the loin was cut for shear force (70 mm) analysis and vacuum-packed, frozen and stored at −20 °C prior to analysis. On analysis, the sample was thawed overnight in its vacuum bag at 4 °C and then cooked in a circulating water bath at 80 °C to an internal temperature of 78 °C, cooled and held on ice overnight. From each cooked section, 10 replicate blocks (20 × 10 × 10 mm) were cut parallel to the fibre direction and sheared with Volodkevitch jaws on a Stevens CR texture analyser (C. Stevens & Son Ltd., St. Albans, Hertfordshire, UK) to give a measure (kg) of shear force (i.e. tenderness).

### Chemical analysis

Both forage (pasture and silage) and meat samples were lyophilized and ground prior to chemical analysis. For vitamin E, all samples were analysed by HPLC as described by Arnold et al. (1993) using 5,7-dimethyl-tocol as an internal standard. Fatty acids in forage were extracted and methylated from 0.5 g of freeze-dried sample using an acidic transesterification described by Sukhija and Palmquist (1988). Fatty acids in meat were bi-methylated directly to fatty acid methyl esters and analysed by gas liquid chromatography on a CP-Select chemically bonded for fatty acid methyl ester column (100 m × 0.25 mm ID, Varian Inc, California, USA) as described by Lee et al. (2012). Identification of fatty acids was performed using external standards and quantified using an internal standard (C23:0) with Varian Star v.6.41 software to capture and handle data.

For mineral assessment, freeze-dried and ground forages and meat (0.5 g sub-samples) were digested with 25 ml nitric/perchloric acid in open tubes. Digested samples were analysed using inductively coupled plasma optical emission spectroscopy to determine concentrations of: aluminium (Al), Ca, iron (Fe), K, magnesium (Mg), Na, P and sulphur (S); and inductively coupled plasma MS to determine concentrations of arsenic (As), cadmium (Cd), Co, chromium (Cr), Cu, Mn, molybdenum (Mo), nickel (Ni), lead (Pb), selenium (Se), titanium (Ti) and Zn. Limits of detection (LOD) were calculated as three times the SD of the reagent blanks and limits of quantification as ten times these SD. A certified reference material (ERM-BB184 Bovine muscle; JRC, Geel, Belgium) was also analysed alongside the samples to determine analytical recoveries, which were within the required range set by the laboratory.

### Statistical analysis of results

For all the variables analysed, both in forages and meat, a randomized complete block design was used. Thus, data were subjected to an ANOVA with pasture system (PP vs. GWC vs. MG) as the treatment, and year (2015, 2016 and 2017) as the block (replicates). For each year and system, one mean value was obtained by averaging across the sub-samples taken throughout the season, i.e. monthly forage samples (silage or pasture) and meat samples from the five steers slaughtered each year from each system. Variance was recorded in the tables as the SED of means. Least significant differences of mean test were used post-ANOVA with significance stated at the *P* < 0.05 level, whereas a trend was defined as *P* < 0.1. All statistical operations were performed in Genstat ® 20th Edition (VSNi, Hemel Hempstead, UK). Observe that for a randomized complete block design, only the main effects can be estimated. Thus, for this study, we are confined to the system means (averaged across the years) and the year effects (averaged across systems). Although the individual year × system means can be calculated, there is no measure of precision (variance) for them, entailing that any statistical comparison is invalid (see our commentary on the statistical methodology used in Supplementary Material S1).

The mean percentage recommended daily intake (RDI) of key nutrients delivered by 100 g of meat from each system was calculated from published values for the RDI for an adult male (BNF, 2016) representing a group of selected nutrients (protein, monounsaturated fatty acids (MUFAs), long chain omega-3 polyunsaturated fatty acids (**PUFAs**), B-vitamins (2, 9 and 12), Ca, Fe, Se, and Zn) as described by Saarinen et al. (2017) and modified by McAuliffe et al. (2018a). The respective carbon footprint per unit of RDI (kg CO_2_-eq/% RDI) was derived as the ratio between RDI units delivered each year (based on a mean 68.5% meat yield per carcass weight) and mass-based carbon footprints of the three systems (21.2, 22.6 and 23.5 kg CO_2_-eq/kg final liveweight for GWC, PP and MG, respectively). These carbon footprint values encompass previously defined greenhouse gas emissions attributable to the suckler enterprise (27.1 kg CO_2_-eq/kg weaning weight: Takahashi et al., 2019) as well as respective finishing enterprises (16.0, 18.5 and 20.2 CO_2_-eq/kg postweaning liveweight gain for GWC, PP and MG, respectively: McAuliffe et al., 2018b). These ratios were calculated on an individual animal basis and then averaged per year within each system. They were analysed via the same general ANOVA as described above for meat and forage variables, along with the other production (weights, day to slaughter, kill out percentage) and quality parameters (fat class, shelf life and tenderness).

Tenderness was only assessed in 2015 and 2016 due to closure of the relevant meat quality laboratory prior to the end of the trial. Fat class scores were converted to a linear scale prior to analysis and then mean values re-categorized post-statistical analysis.

## Results

### Forage composition – Silage

Silage fatty acid concentrations and their differences across the systems are reported in Table 1, where only three FAs significantly varied among farmlets; C12:0 was higher for PP and differed from GWC and MG silages, C16:0 was higher for PP and MG silages, compared to that of GWC systems, whereas the MUFA C18:1n-9 was higher in MG silage, lower in PP, and intermediate in the GWC silage (not differing from the other two). The saturated fatty acid (SFA) C14:0 showed a trend for higher concentrations in PP and lower in GWC. The SFAs C18:0 and C20:0 showed no difference across systems averaging at 0.36 and 0.15 g/kg DM, respectively. Regarding MUFAs, C16:1n-7 and C18:1n-7 did not vary among systems, averaging at 0.21 and 0.14 g/kg DM, respectively. Similarly, the PUFAs C18:2n-6 and C18:3n-3 did not vary among systems, averaging at 3.14 and 9.51 g/kg DM, respectively. Likewise, the sum of FAs was similar between systems averaging at 21.4 g/kg DM.

**Table 1:** Fatty acids (**FAs**), vitamin E and mineral composition of the silage offered to the cattle during winter housing from three pasture-based systems over 3 years.

Item	PP	GWC	MG	SED	*P* (F_2,4_)
Fatty acids (g/kg DM)					
C12:0	0.100^a^	0.082^b^	0.080^b^	0.0039	**0.014**
C14:0	0.203	0.165	0.183	0.0110	*0.062*
C16:0	3.61^a^	3.21^b^	3.65^a^	0.113	**0.033**
C16:1n-7	0.222	0.175	0.224	0.0211	0.131
C18:0	0.367	0.344	0.380	0.0155	0.170
C18:1n-9	0.553^b^	0.600^ab^	0.648^a^	0.0231	**0.037**
C18:1n-7	0.145	0.135	0.152	0.0104	0.361
C18:2n-6	3.21	2.91	3.31	0.242	0.335
C18:3n-3	10.1	8.59	9.87	0.816	0.257
C20:0	0.148	0.147	0.157	0.0053	0.234
Sum FAs	22.6	19.5	22.4	1.32	0.133
Vitamin (mg/kg DM)					
E	71.3	76.2	86.7	4.63	*0.067*
Macro minerals (g/kg DM)					
Ca	7.115	7.694	7.001	0.7827	0.666
K	25.0^b^	24.3^c^	26.7^a^	0.47	**0.016**
Mg	1.87^a^	1.62^b^	1.57^b^	0.078	**0.037**
Na	2.95^a^	1.79^b^	1.82^b^	0.235	**0.013**
P	3.20	3.05	3.12	0.116	0.498
S	2.24	2.04	2.25	0.103	0.183
Micro minerals (mg/kg DM)					
As	0.251	0.239	0.253	0.0333	0.912
Co	0.824	0.654	0.750	0.1816	0.671
Cr	1.87	1.89	2.77	0.545	0.277
Cu	24.7	20.6	22.2	3.15	0.491
Fe	487	472	475	71.0	0.974
Mo	1.36	1.11	0.96	0.182	0.204
Mn	182^a^	156^b^	140^b^	9.6	**0.030**
Ni	1.35	1.44	1.79	0.128	*0.056*
Se	0.247	0.231	0.243	0.0302	0.857
Zn	70.6	51.8	59.4	11.91	0.377
Non-nutritional elements (mg/kg DM)					
Al	544	550	543	91.4	0.996
Cd	0.036	0.039	0.042	0.0057	0.629
Pb	0.345	0.356	0.316	0.0757	0.867
Ti	11.0	10.4	10.9	0.6764	0.661

Abbreviations: PP = permanent pasture; GWC = grass and white clover; MG = short-term monoculture grass ley.

^abc^Values within a row with different superscripts differ significantly at *P* < 0.05.

There was a trend for Vitamin E concentration in the silages from the pastures to be higher in MG and lowest in PP. Of the macro minerals, Ca, P and S were not significantly different across treatments averaging at 7.27, 3.12 and 2.18 g/kg DM, respectively. K was highest in MG silage, intermediate in PP and lowest in MG, while Mg and Na were both highest in PP silage, with no difference between GWC and MG. Concerning the micro minerals, Mn differed among farmlets, being highest in PP and lowest in GWC and MG. Ni tended to be highest in MG and lowest in PP and GWC. As, Co, Cr, Cu, Fe, Mo, Se and Zn all showed no difference across silages. Regarding the non-nutritional elements, there were no significant differences across the three silages. The variation of the nutritional composition of the silage fed between years is shown in Supplementary Table S1.

### Forage composition – Grazed pasture

The fatty acid profiles of the grazed pasture did not significantly vary among the three systems (Table 2). Sum of FAs did not vary either and averaged at 33.2 g/kg DM. Similarly, vitamin E was not different across pasture systems averaging at 36.2 mg/kg DM.

**Table 2:** Fatty acids (FAs), vitamin E and mineral composition of the pasture grazed by the cattle from three pasture-based systems over 3 years.

Nutrient	PP	GWC	MG	SED	*P* (F_2,4_)
Fatty acids (g/kg DM)					
C12:0	0.123	0.105	0.102	0.0157	0.431
C14:0	0.180	0.153	0.145	0.0156	0.182
C16:0	4.79	4.84	4.79	0.283	0.977
C16:1n-7	0.057	0.057	0.059	0.0054	0.932
C18:0	0.508	0.555	0.518	0.0514	0.655
C18:1n-9	0.625	0.658	0.651	0.0876	0.926
C18:1n-7	0.111	0.108	0.109	0.0042	0.797
C18:2n-6	3.38	3.30	3.35	0.384	0.978
C18:3n-3	17.6	16.0	16.1	1.63	0.599
C20:0	0.167	0.172	0.169	0.0063	0.753
Sum FAs	33.0	31.2	31.4	2.59	0.742
Vitamin (mg/kg DM)					
E	33.1	40.4	35.1	5.98	0.509
Macro minerals (g/kg DM)					
Ca	5.75^b^	7.81^a^	4.72^b^	0.554	**0.012**
K	28.2	29.1	29.8	1.51	0.644
Mg	1.73^a^	1.75^a^	1.40^b^	0.067	**0.010**
Na	1.70^b^	0.72^b^	0.80^a^	0.218	**0.019**
P	3.76	3.64	3.54	0.116	0.285
S	2.93	2.88	2.89	0.137	0.919
Micro minerals (mg/kg DM)					
As	0.096	0.153	0.164	0.0222	*0.073*
Co	0.058	0.173	0.135	0.0370	*0.081*
Cr	0.915	1.26	1.41	0.2230	0.193
Cu	8.41^a^	7.70^b^	7.13^b^	0.252	**0.018**
Fe	154	311	270	49.1	*0.071*
Mo	2.04	1.38	1.27	0.370	0.218
Mn	120	105	106	10.5	0.361
Ni	1.19	1.48	2.03	0.404	0.223
Se	0.069	0.063	0.071	0.0082	0.630
Zn	27.4^a^	23.8^b^	24.0^c^	0.69	**0.011**
Non-nutritional elements (mg/kg DM)					
Al	170	406	382	73.0	*0.058*
Cd	0.024	0.037	0.034	0.0070	0.257
Pb	0.161^b^	0.341^a^	0.282^a^	0.0315	**0.011**
Ti	6.61	9.15	8.33	0.948	0.121

Abbreviations: PP = permanent pasture; GWC = grass and white clover; MG = short-term monoculture grass ley.

^abc^Values within a row with different superscripts differ significantly at *P* < 0.05.

Macro minerals Ca, Mg and Na significantly differed among pasture systems; Ca was highest in GWC and lowest in the other two pasture types; Mg was highest in PP and GWC and lowest in MG; and Na was highest in MG and lowest in the other two pastures. Conversely, K, P and S did not vary among pasture systems averaging at 29.1, 3.65 and 2.90 g/kg DM, respectively. For the micro minerals, only Cu and Zn significantly differed between systems; Cu was highest in PP and lowest in the other two systems, Zn was highest in PP, intermediate in GWC and lowest in MG. For trends (*P* < 0.1), As, Co and Fe tended to be highest in GWC and MG. The remaining micro minerals displayed no variation among pasture types. Regarding non-nutritional elements, only Pb significantly varied with pasture types, being lowest in PP. Al tended to be lowest in PP, whereas Cd and Ti were comparable across treatments averaging at 0.032 and 8.03 mg/kg DM, respectively. The variation of the nutritional composition of the grazed pasture between years is shown in Supplementary Table S1.

### Performance traits

Results for performance traits are given in Table 3, where weaning weight, slaughter weight and live weight gain from weaning to slaughter did not significantly vary among systems averaging at 323, 665 and 346 kg, respectively. Similarly, days to slaughter and body condition score were similar among systems, averaging at 885 d and 4.01, respectively. Carcass weight, fat score and kill out percentage were also similar among systems averaging 337 kg, 4.62 (which converts to 4L), and 52.1%, respectively. Tenderness (2015–2016 only) and RDI delivery of selected nutrients similarly produced no significant differences across treatments, averaging at 4.88 kg and 29.3%, respectively. Shelf life of the matured steaks tended to be longest for PP and shortest for GWC. Carbon footprint per unit of RDI delivery was significantly lower on GWC than the other two treatments, with no difference between PP and MG. For useful context, the annual meteorological conditions for the NWFP for each year of forage production indicating turnout, silage cut and finishing are shown in Supplementary Figure S1.

**Table 3:** Performance traits of the cattle (*n* = 15) from the three pasture-based systems over 3 years.

Variable	PP	GWC	MG	SED	*P* (F_2,4_)
Weaning weight (kg)	328	324	317	7.0	0.352
Slaughter weight (kg)	662	674	658	27.5	0.844
Days to slaughter	643	656	661	16.5	0.573
LWG (kg)	348	348	341	18.6	0.909
Body condition score	4.07	4.00	3.95	0.155	0.766
Fat score	4.73	4.73	4.40	0.455	0.720
Carcass weight (kg)	346	348	344	13.11	0.954
Kill out (%)	52.2	51.7	52.4	0.48	0.433
Shelf life (days of RD)	13.1	11.5	12.3	0.47	*0.068*
Tenderness (kg)	4.94	4.86	4.84	0.139	0.744
RDI (%/100 g FW)	29.1	29.2	29.4	0.22	0.409
kg CO_2_e/RDI%	0.215^a^	0.191^b^	0.231^a^	0.0079	**0.018**

Abbreviations: PP = permanent pasture; GWC = grass and white clover; MG = short-term monoculture grass ley; LWG = liveweight gain at pasture; RD = Retail display; RDI = recommended daily intake; FW = Fresh weight; CO_2_e = Carbon dioxide equivalents.

^ab^Values within a row with different superscripts differ significantly at *P* < 0.05.

### Meat composition – Fatty acids and vitamin E

The fatty acid compositions (mg/100 g Fresh matter) of the beef steaks are presented in Table 4 and Supplementary Table S2 (percentage of total FAs). When expressed as composition, the only FAs that differed among farmlets were the PUFAs C18:2n-6 and C20:3n-6, and the sum of omega-6 PUFAs; GWC showed the highest values whilst PP and MG showed the lowest values. Additionally, the PUFA C22:4n-6 tended to be highest in GWC and lowest in the other two systems; consequently, sum of PUFAs tended to be highest in GWC compared with the other two systems. The remaining FAs or groups of FAs did not differ among systems (Table 4), nor the percentage or ratios of any FA or group of FAs (Supplementary Table S2).

**Table 4:** Fatty acids (FAs) and vitamin E composition of the *Longissimus dorsi* of the cattle from the three pasture-based systems over 3 years.

	PP	GWC	MG	SED	*P* (F_2,4_)
Fatty acids (mg/100 g FM)					
C12:0	1.54	1.75	1.52	0.179	0.437
C14:0	57.9	68.3	58.3	8.37	0.446
C15:0	13.9	14.2	15.0	1.83	0.815
C16:0	614	705	609	84.7	0.509
C16:1n-7	86.8	98.2	82.4	10.81	0.405
C17:0	29.1	31.2	30.1	3.31	0.822
C18:0	367	386	372	55.4	0.944
C18:1n-9	933	1061	909	141.1	0.563
C18:1n-7	31.0	35.5	28.9	2.90	0.182
C18:1 trans 6 + 7 + 8 + 9	7.04	7.41	6.72	1.19	0.848
C18:1 trans 10 + 11	51.6	44.7	45.8	10.21	0.780
C18:2 CLA	14.0	13.5	11.6	2.65	0.679
C18:2n-6	51.1^b^	57.3^a^	48.6^b^	1.96	**0.025**
C18:3n-3	29.8	31.5	25.9	2.95	0.269
C20:1n-9	3.16	3.79	3.05	0.518	0.388
C20:3n-6	5.26^ab^	6.04^a^	4.92^b^	0.299	**0.045**
C20:4n-3	4.39	4.63	3.95	0.385	0.305
C20:4n-6	19.6	20.8	19.7	0.77	0.344
C20:5n-3	16.3	15.2	15.7	0.64	0.337
C22:4n-6	1.32	1.56	1.26	0.092	*0.065*
C22:5n-3	24.0	24.8	22.5	0.92	0.140
C22:6n-3	3.32	2.96	3.15	0.192	0.292
Sum FAs	2599	2871	2546	339.1	0.626
Sum SFAs	1041	1160	1041	144.0	0.662
Sum MUFAs	1112	1250	1076	163.2	0.577
Sum n-3 PUFAs	77.9	79.1	71.2	4.41	0.269
Sum n-6 PUFAs	77.3^b^	85.7^a^	74.4^b^	2.39	**0.021**
Sum PUFAs	155	165	146	6.27	*0.090*
Vitamin (mg/kg DM)					
E	5.14	4.39	4.96	0.335	0.177

Abbreviations: PP = permanent pasture; GWC = grass and white clover; MG = short-term monoculture grass ley; FM = Fresh material; CLA = conjugated linoleic acid; SFAs = saturated fatty acids; MUFAs = monounsaturated fatty acids; PUFAs = polyunsaturated fatty acids.

^ab^Values within a row with different superscripts differ significantly at *P* < 0.05.

### Meat composition – Minerals

Other than for one sample on the PP treatment in 2015, Ca and Al were all below the LOD, as were As and Co for all samples, meaning that no outcomes could be reported for these minerals (Table 5). Regarding the macro minerals, Mg and Na significantly varied with treatment, where PP and MG had the highest concentrations, respectively. K, P and S were not significantly different between treatments, with average values of 363, 177 and 171 mg/100 g FM, respectively. Similarly, all micro minerals and all non-nutritional elements were not significantly different between treatments.

**Table 5:** Mineral composition of the *Longissimus dorsi* of the cattle (*n* = 15) from the three pasture-based systems over 3 years.

Element	PP	GWC	MG	SED	*P* (F_2,4_)
Macro minerals (mg/100 g FM)					
Ca	BDL	BDL	BDL		
K	363	361	364	2.7	0.581
Mg	23.3^a^	23.0^b^	23.4^a^	0.05	**0.004**
Na	46.9^b^	48.5	49.5^a^	0.68	**0.049**
P	179	175	177	2.8	0.435
S	173	169	171	2.7	0.343
Micro minerals (mg/100 g FM or μg/100 g FM^β^)					
As	BDL	BDL	BDL		
Co	BDL	BDL	BDL		
Cr^β^	25.5	27.3	18.7	18.71	0.847
Cu^β^	60.5	57.8	64.1	2.83	0.203
Fe	1.91	1.96	20.3	0.129	0.686
Mo^β^	0.59	0.58	0.56	0.097	0.963
Mn^β^	10.8	10.07	10.9	0.75	0.534
Ni^β^	7.92	4.81	8.35	3.057	0.511
Se^β^	5.13	6.70	7.01	0.830	0.162
Zn	4.04	3.91	4.03	0.113	0.522
Non-nutritional elements (mg/100 g FM or μg/100 g FM^β^)					
Al	BDL	BDL	BDL		
Cd^β^	0.575	0.182	1.173	0.5481	0.299
Pb^β^	3.38	2.86	13.5	5.35	0.196
Ti^β^	81.3	79.7	81.1	1.28	0.462

Abbreviation: PP = permanent pasture; GWC = grass and white clover; MG = short-term monoculture grass ley; FM = fresh matter; BDL = below detection limit. Elements close to their analytical limit of detection (LOD): Al, LOD 3.65 mg/100 g FM; As, LOD 1.1 μg/100 g FM; Ca, LOD 8.32 mg/100 g FM; Cd, LOD 0.08 μg/100 g FM; Co, 7.6 μg/100 g FM; Cr, LOD 3.7 μg/100 g FM; Mo, LOD 0.26 μg/100 g FM; Ni, LOD 1.6 μg/100 g FM; Pb, LOD 1.3 μg/100 g FM.

^ab^Values within a row with different superscripts differ significantly at *P* < 0.05.

## Discussion

### Pasture composition

The differences observed for fatty acids between pastures were small, with year (block) having a greater effect than treatment (Supplementary Table S1). This is consistent with the findings of Elgersma et al. (2003) who investigated changes in fatty acid profiles of perennial ryegrass and showed significant differences during defoliation regime related to growth conditions and leaf:stem ratio. Furthermore, significant differences in fatty acid content and profile have been observed across seasons, with highest values in spring and autumn then reducing in the summer (Bauchart et al., 1984). Moloney et al. (2018) reported similar results for fatty acid profiles and total fatty acid content of grass and grass/white clover swards grazed by beef cattle, with a mean across treatments and years of 31.7 g/kg DM.

Elgersma et al. (2003) reported a significant drop in all fatty acids but especially PUFA between fresh and ensiled forage due to oxidative loss, microbial metabolism and postlipolytic exudative loss. The same pattern between pasture sampled at grazing and silage sampled as fed was observed in the current study, with 32% and 42% reduction for total fatty acids and C18:3n-3, respectively. GWC silage had lower levels of the major C16 fatty acid than either PP or MG but was comparable for all other fatty acids, whereas, Lee et al. (2003) reported higher levels of most fatty acids, including the major SFA, C16:0 in white clover silage compared to perennial ryegrass silage. The current finding may relate to the available N to the sward at the time of first cut silage, as N is typically released later in the season for legume N-fixation swards (Palmer and Iverson, 1983). Fertilizer N has been shown, through enhanced foliar growth, to significantly increase pasture fatty acid content (Boufaïed et al., 2003).

Vitamin E concentration within forage can be highly variable, ranging between 9 and 400 and 0 and 310 mg/kg DM for fresh and ensiled forage, respectively, as reported in the review of Ballet et al. (2000). The wide range for both fresh and ensiled forage relates to differences between forage species, climatic growing conditions and stage of maturity, as well as quality of the conservation methodology for the latter (Ballet et al., 2000). Beeckman et al. (2009) also reported a large variation in vitamin E for grass/clover silage with a mean of 52 mg/kg DM, which is comparable with our current findings. However, Beeckman et al. (2009) reported levels in fresh grass (156 mg/kg DM) which was higher than their average silage value, contrary to the present study. They also reported fresh white clover to have lower values than grass at 49 mg/kg DM, a finding supported by the review of Ballet et al. (2000) which states that, at early stages of growth, grasses typically have twice the level of vitamin E than legumes, whereas at the flowering stage, levels are comparable. In the current study, the MG silage tended to have a higher vitamin E than PP and GWC, with no differences between the grazed pastures. These results likely relate to differences in sampling methods between the studies: Beeckman et al. (2009) sampled pasture to simulate a silage cut, whereas in the present study, forage was sampled at grazing. This would significantly alter the leaf:stem ratio, with vitamin E significantly higher in leaf than stem material (Ballet et al., 2000). This factor may also partly explain the higher levels of vitamin E observed in silage than fresh grass in the present study, although this result is predominately due to the sampling of silage as fed, inclusive of postsupplemental vitamin and mineral addition.

The NWFP performs regular soil sampling surveys, with all data including mineral contents and soil conditions (e.g. organic matter, pH, redox potential = Eh) freely available on the National Capability’s data portal (http://resources.rothamsted.ac.uk/farmplatform). Using this dataset for the same PP, GWC and MG treatments assessed herein, Kao et al. (2020) showed that total soil mineral concentration poorly described the mineral status of forage, with weak coefficients of determination (*R*^2^ < 0.01) between soil and forage for Cu, Zn, Fe and Mn. The available pool of minerals in soils is regulated by several chemical and biological processes, including plant removal, sorption–desorption from sorption sites, complexation with inorganic/organic complexes, precipitation, micro-organism activity and leaching/surface runoff (Bolan et al., 2004, Kao et al., 2020). Soil pH and Eh are the two major variables for the geochemical processes controlling the solubility and mobility of minerals in soil (Bourg and Loch, 1995). These variables are likely to be the main factors driving differences in the reported pasture mineral content observed here, e.g. higher Zn and Cu associated with PP swards, which may reflect their uncultivated status. Kao et al. (2020) reported that in most cultivated soils, the availability of Cu and Zn is more likely to be affected by a change of pH than Eh, whereas for Fe, Mn, Co and Se, the variation of pH and Eh equally influence their chemical speciation.

The concentration of minerals reported in the grazed pastures of the current study agrees with the range provided for typical forage grown in temperate UK conditions, except for Mn, which was higher than the range of 40–80 mg/kg DM reported by Lee et al. (2018). This may reflect the dominance of perennial ryegrass within the swards (Orr et al., 2016), as grass has been shown to exhibit higher concentrations of Mn than legumes or forbs (Lindström et al., 2013). The mineral concentration of the silage was higher than that of the grazed pasture, especially in terms of the micro minerals and the non-nutritional element Al. This increase is likely a result of three main factors: (i) loss of organic matter during ensiling resulting in a concentration of minerals; (ii) silages being sampled as fed, which included the addition of the mineral premix into the mixer wagon – hence explaining the greater effect in micro minerals supplemented (Co, Cu, Mn, Zn and Se); and (iii) soil contamination during silage harvesting – this would explain the elevated levels of not only micro minerals but also Al (Mayland et al., 1977). Mineral intake from soil ingestion either at grazing or from contaminated silage can significantly contribute to mineral intake of ruminant livestock (Kao et al., 2020). However, availability of minerals consumed via this route has been shown to be low due to their form within soil as opposed to when taken up by plants (Healy et al., 1970).

### Animal performance and meat composition

Animal performance in terms of live weight gain, slaughter weights and killing out percentages were not different between treatments and comparable to previously reported values for cattle finished off silage and pasture (Warren et al., 2008a) or grazed pasture with or without white clover (Moloney et al., 2018). Fat scores were not different across treatments and averaged to the equivalent of 4L as targeted.

Tenderness of the beef was not different between treatments and was comparable to publications reviewed by Moloney et al. (2001), who compared the proportion of forage in the diet and tenderness score and found no difference between diets. Shelf life is an important quality trait and is driven by the antioxidant capacity (vitamin E and the seleno-enzyme glutathione peroxidase) of the meat and the PUFA content which increases the oxidative stress on the meat. In the current study, shelf life was higher (11.5–13.1 days) than that previously reported for pasture-based beef by Warren et al. (2008b) at 7 days, which may reflect the lower vitamin E content of their meat (3.2 mg/kg DM) compared with an average of 4.8 mg/kg DM in the current study. The trend to have higher shelf life of PP compared with GWC in the current study may relate to a trend for lower PUFA in the PP meat than the GWC meat, with comparable vitamin E concentrations. Although Lee et al. (2019) showed that Se was positively correlated with shelf life in lamb when vitamin E levels were comparable, Ripoll et al. (2011) reported that vitamin E expressed a greater level of oxidative protection over Se. This also appears to be the case in the current study, as no clear pattern of Se in muscle related to shelf life was observed.

The fatty acid profile of the meat as well as appearance of trans MUFA and conjugated dienes such as conjugated linoleic acid is a consequence of ruminal lipid metabolism and the rumen-microbial process of biohydrogenation (Warren et al., 2008a). The impact of these mechanisms on fatty acid profiles has extensively been discussed in the literature and is reviewed by Daley et al. (2010) in respect to high forage diets. The fatty acid composition of the meat and the pattern between treatments in this study are highly comparable to the findings of Moloney et al. (2018), who also reported higher concentrations of C18:2n-6 and total n-6 PUFA on grass and white clover finished cattle than grass finished cattle. Higher DM intake increasing substrate supply or altered rumen digestion kinetics with a greater flow of PUFA across the rumen in white clover containing diets (compared to sole grass diets) may offer potential explanation (Lee et al., 2003), although neither mechanism was assessed in the current study. Despite these small but significant differences in PUFA content, overall, the impact of pasture treatment on fatty acid profile was small.

The mineral profile of beef is affected by a wide range of intrinsic factors such as muscle type (Czerwonka and Szterk, 2015), animal breed (Valaitienė et al., 2016, Patel et al., 2019), sex and age (Giuffrida-Mendoza et al., 2007, Schönfeldt and Hall, 2015); extrinsic factors such as diet (de Freitas et al., 2014, Schönfeldt and Hall, 2015) and processing protocol (Ramos et al., 2012) also play considerable roles. The only difference observed in mineral concentrations of beef across the three pastures was for numerically significant elevations, but biologically small differences, in Mg and Na for PP and MG finished beef, respectively, which are likely related to intake differences (Mg was higher in PP silage and Na was higher in MG pasture). All other minerals were not different between treatments with values comparable to those reported in pasture-based studies by Patel et al., 2019, Valaitienė et al., 2016, de Freitas et al., 2014, except for K, Mn and Ti which were higher in the current study than those three studies. These studies did not report values for Mo, Cd and Pb, which were detected in the current study (nor Ca, As, Co and Al, although these were predominately below the limit of detection in the current study). The differences observed for K and Mn are likely to be associated with dietary differences between studies. As already reported, K was applied annually to each treatment and Mn is typically higher in grass dominated swards. Few studies have reported Ti levels in meat: Patel et al. (2019) reported 1.4 μg Ti/100 g meat from cattle on forage-based diets in Italy, whereas 68.2 μg Ti/100 g meat was found in a study with Dexter cattle raised solely from forage in the UK (S. Morgan personal communication), which aligns more with the current study and is likely related to soil differences. Heavy non-nutritional elements in meat, such as Pb and Cd, are thought to be diet related, ultimately driven by levels in soil. Values observed in beef tissue in the current study were below upper limits for Pb (10 μg/100 g) (EFSA, 2010) and Cd (0.60 μg/100 g) (González-Weller et al., 2006).

### Nutritional value and the role of pasture beef in sustainable diets

The nutritional value of food primarily relates to the key bioavailable nutrients it contains and their densities are indicative of the amounts that should be consumed based on the RDI. However, the environmental impact of a food is typically assessed by a functional unit linked to mass of product (g meat) or unit of a single nutrient (g protein), failing to recognize the true function of food for human health (McAuliffe et al., 2020). When the average RDI across 10 essential nutrients was computed to address this issue, no significant difference was observed between treatments (Table 3); this result was unsurprising given the similar contents of key fats and minerals between the three systems studied. When this value was further combined with carbon footprint of the respective system to represent the climate impact of pasture-based beef relative to its critical nutritional value, GWC was shown to produce significantly less CO_2_-eq per unit of RDI than the other pasture-based systems, predominantly due to lower use of inorganic fertilizer (McAuliffe et al. 2018b). With the ever-growing societal need to balance environmental and health performances of agri-food systems, these approaches are likely to shape the future form of sustainability assessments to derive strategies that can simultaneously deliver human and planetary health. As such, the results presented here can be used as an evidence base to assist comparisons with non-pasture-based beef systems, livestock species and plant-based alternatives on a like-for-like basis.

As a wider assessment of the nutritional value of pasture beef, Table 6 sets out RDI requirements for a wider group of key nutrients (protein, fat, minerals and vitamins) for an average male adult alongside the nutrient delivery from 100 g of lean temperate pasture-based beef. Primary data from the current study were used where available (as means across treatments), with values from the literature obtained in the absence of primary data (see Table 6 for a list of sources). On average and across all key nutrients (27), it was shown that a 100 g serving of beef provides 18.6% of RDI values. As, Cr, Mn and Ni, for which there is a nutritional requirement but no RDI has been set (Stergiadis et al., 2019), were not included in the present assessment. Pasture-based beef was shown to be a high source (defined as >20% RDI per serving) of protein, MUFA, SFA, vitamins – B2, B3, B12 and minerals – Fe, P, Zn; a good source (10–19% RDI per serving) of vitamin – B6 and mineral – K; and a complementary source (5–9% RDI per serving) of omega-3 fatty acids, long chain omega-3 fatty acids, vitamin – B9 and minerals – Cu, Mg, Se.

**Table 6:** Recommended Daily Intake of Protein, Fat, Vitamins and Minerals and their provision from 100 g FM serving of pasture-based beef.

	Units	RDI (d^−1^)	Beef (100 g FM)	RDI %
Protein	g	50.3^a^	23.5^b^	46.8
Fat				
Omega-3	g	1.60^c^	0.081	5.03
Omega-6	g	8.00^c^	0.078	0.97
MUFA	g	3.75^b^	1.15	30.8
Long chain Omega-3	mg	250^b^	18.9	7.57
SFA	g	1.50^c^	1.08	43.1
Vitamins				
β-carotene (A)	mg	3.00^d^	0.07^e^	2.47
B1	mg	0.80^a^	0.03^f^	3.75
B2	mg	1.10^a^	0.26^b^	23.6
B3	mg	12.0^a^	4.58^f^	38.2
B6	mg	1.20^a^	0.20^f^	16.7
B9	μg	200^a^	16.0^b^	8.00
B12	μg	1.50^a^	2.00	133
C	mg	90.0^a^	–	–
D	μg	10.0^a^	0.40^f^	4.00
E	mg	15.0^a^	0.48	3.20
K	μg	120	1.70	1.42
Minerals				
As*	μg	36.0^g^	–	–
Ca	mg	700^a^	5.00^b^	0.71
Cr*	μg	35.0^g^	22.7	64.9
Cu	mg	1.20^a^	0.06	5.00
Fe	mg	8.70^a^	1.96	22.5
K	g	3.50	0.36	10.3
Mg	μg	300	23.2	7.74
Mn*	mg	2.30^g^	0.01	0.47
Mo	μg	45.0^g^	0.59	1.31
Na	g	1.60^a^	0.05	3.03
Ni*	μg	140^g^	6.56	4.70
P	mg	550^a^	177	32.2
Se	μg	75.0^a^	6.10	8.13
Zn	mg	9.50^a^	3.99	42.0

Abbreviations: FM = Fresh material; RDI = Recommended Daily Intake for an Adult male; MUFA = Monounsaturated Fatty Acid; SFA = Saturated Fatty Acid; Long chain omega-3 = C20:5n-3 + C22:6n-3.

* RDI not available, average daily intake reported.

a Values from the literature are denoted by a superscript: BNF (2016).

b McAuliffe et al. (2018a).

c EFSA (2009).

d NIHCC (2002), Ref Type: Pamphlet.

e Daley et al. (2010).

f http://www.freenutritionfacts.com/beef/vitamins/.

g IoM (2001).

Finally, it is important to note that chemical contents of nutrients in food do not directly translate to human health, as the body’s capability to utilize them also depends on cooking losses, digestibility, metabolizability and bioavailability of these nutrients, as well as the complementarity between them and the health and metabolic status of the consumer. This is a particularly important consideration for protein, which greatly differs in essential amino acid composition and digestibility depending on the commodity through which it is consumed. In this regard, case studies using the digestible indispensable amino acid score (DIAAS) have shown that animal-based proteins, when not processed, typically have the highest values of all food groups (Marinangeli and House, 2017).

Combined, the above results suggest that pasture-based beef has a high nutritional value and, when consumed responsibly, can constitute an important component of a healthy balanced diet. Furthermore, given that the overall nutritional quality of meat was found to be largely comparable across all three temperate pasture treatments from the present study, this conclusion is thought to be transferrable to a wide range of forage-based production systems found in temperate regions across the globe.

## Supplementary material

Supplementary data to this article can be found online at https://doi.org/10.1016/j.animal.2021.100257.

## Ethics approval

All animal procedures and the care for the animals were carried out under strict regulations described in the Animals (Scientific Procedures) Act 1986 issued by the Home Office of Her Majesty’s Britannic Government.

## Data and model availability statement

None of the data were deposited in an official repository. The data that support the study findings are available upon request. Please also see the supplementary material.

## Author ORCIDs

**Michael R.F. Lee:** https://orcid.org/0000–0001-7451–5611.

## Author contributions

**MRFL** and **LC** secured the funding. **MRFL**, **PH**, **GAM**, and **TT** designed the study. **GAM**, **SAM** and **BAG** collected the data. **JKST**, **GAM** and **SAM** analysed the data. **MRFL**, **MJR**, **PH** and **TT** prepared the draft. All authors critically reviewed the draft and contributed to the final version of the manuscript.

## Declaration of interest

All authors declare no conflict of interests.
