# Supplementary material for: Nutritional value of suckler beef from temperate pasture systems
Source: Animal. 2021 Jul;15(7):None. doi: 10.1016/j.animal.2021.100257 (PMC8282502; doi:10.1016/j.animal.2021.100257)
Supplement: Supplementary data 1 [file mmc1.doc]

***animal* Journal**

**Nutritional value of suckler beef from temperate pasture systems**

M.R.F. Lee ^a,b,c^, G.A. McAuliffe ^a^, J.K.S. Tweed ^d^, B.A. Griffith ^a^, S.A. Morgan ^a^, M.J. Rivero ^a^, P. Harris ^a^, T. Takahashi ^a,b^. L. Cardenas ^a^

^a^ *Rothamsted Research, North Wyke, Okehampton, Devon, EX20 2SB, UK,*

*^b^ University of Bristol, Bristol Veterinary School, Langford, Somerset, BS40 5DU, UK,* ^c^ *Current address: Harper Adams University, Newport, Shropshire, TF10 8NB*

*^d^ Aberystwyth University, Institute of Biological, Environmental and Rural Science, Gogerddan, Aberystwyth, Ceredigion, SY23 2EB, UK*

**Supplementary Material S1**

***Commentary on the statistical methodology used***

Lack of replication is common in systems studies, and an issue that has been frequently encountered in ecological and other studies, where an argument of pseudo-replication (Hurlbert, 1984) is sometimes made against the publication of such results. For the North Wyke Farm Platform experiment, it can easily be argued that the farmlet is the experimental unit, and as such, an ANOVA is invalid. The experiment is a comparison of systems encompassing all of its processes (soils, plants, animals, emissions etc.), where for an animal-focussed study there can never be spatial replication, as this data can only ever be at the farmlet scale. However, as done in this study, the years can be taken as replicates but with caveats.

It is our view that a pragmatic approach should be taken to questions of this sort where 'physical' replication is difficult or impossible. The question then becomes “Is it possible to make any reasonable inferences from the data?” If the answer is no, then results are not strictly publishable from system-based trials that have no physical replication, which would be unfortunate and, to some extent, unreasonable. How else can one build up a body of data to prove a claim? The view that replication over time should be treated as a repeated measures study has validity, although the definition of repeated measures deserves some scrutiny: in practice, repeated measures refer to sampling or measuring the same experimental unit over time, but there is a whole spectrum of situations where the temporal dependence varies from very strong stepwise dependence to effective independence. In this study, we have (of course) put the dependence quite some way towards the latter. Although the treatments are the same from year to year and the physical unit is the same, its manifestation in terms of the sward characteristics will vary, particularly driven by changes in the weather. The animals, on which the important measures are made are variable from year to year, and there is no way in which they can be truly considered as repeated measures.

As the farmlet is the primary experimental unit - is it the same from year to year? The annual variation of the silage and pasture does not entirely bear this out, and the cattle are not the same, but given this, there is still a reasonable argument for considering years as replicates. Certainly, they are not random, but they are variable, and variation in the weather year to year can be considered as a form of blocking imposed on the three systems. A preliminary assessment of the data suggested that the impact of individual years is reasonably consistent across systems. So, as done in this study, taking the underlying model as a randomised complete block design (RCBD) with years as 'blocks' and systems as 'treatments', an ANOVA could be performed yielding system means (averaged over the three years) and year means (if required) with the interaction providing an estimate of error. The impact of this decision was to inflate the error if there is a large interaction between time and system, but it is how RCBDs are characteristically treated.

Hurlbert, S.H., 1984. Pseudoreplication and the Design of Ecological Field Experiments. Ecological Monographs 54, 187-211.

**Supplementary Figure S1.** The annual meteorological conditions for the North Wyke Farm Platform for each year of forage production for finishing cattle indicating turnout, silage-cut and finishing for 2014, 2015, 2016 and 2017. Abbreviations: PP = permanent pasture; GWC = grass and white clover; GM = short-term monoculture grass ley.


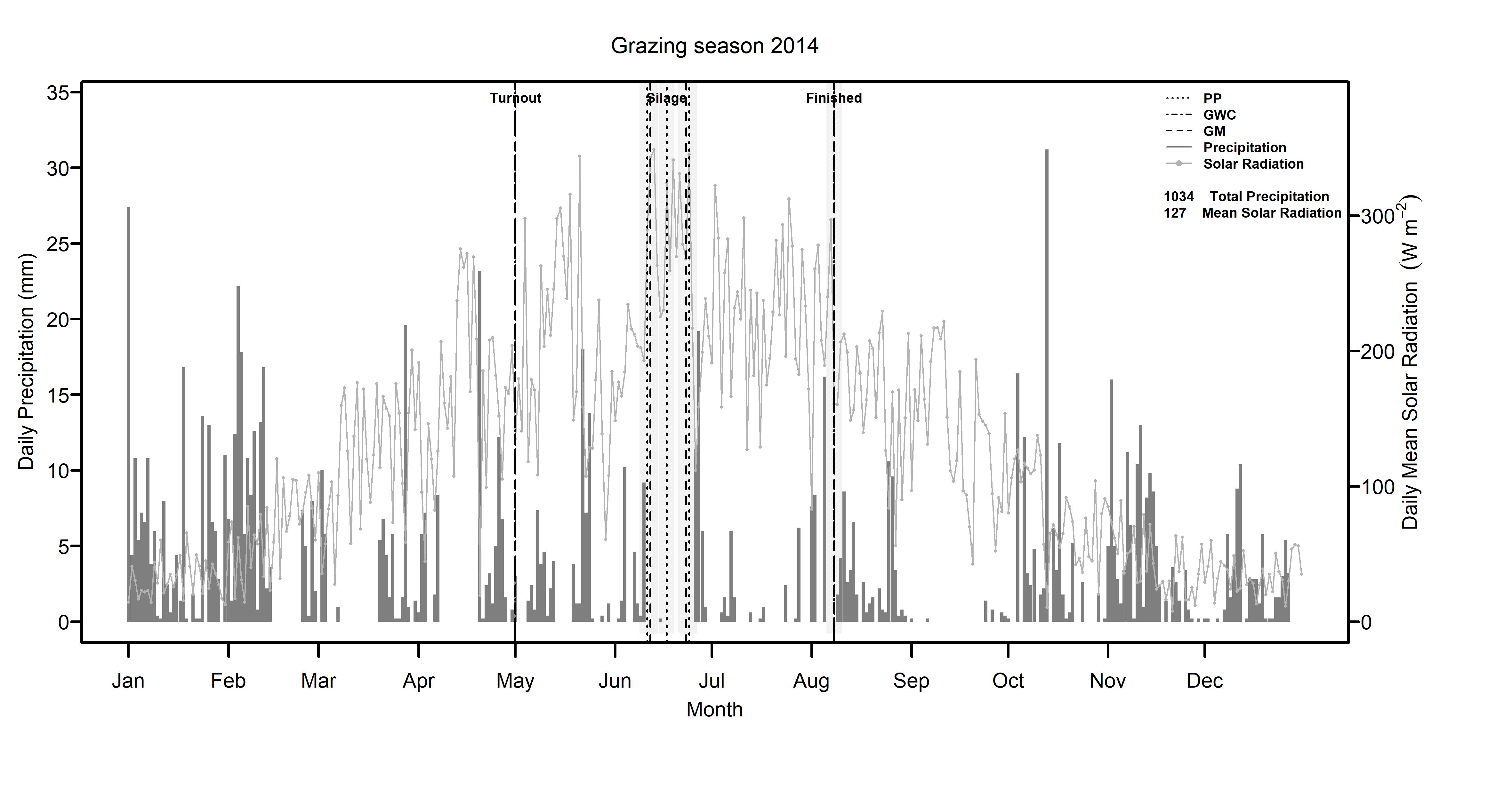


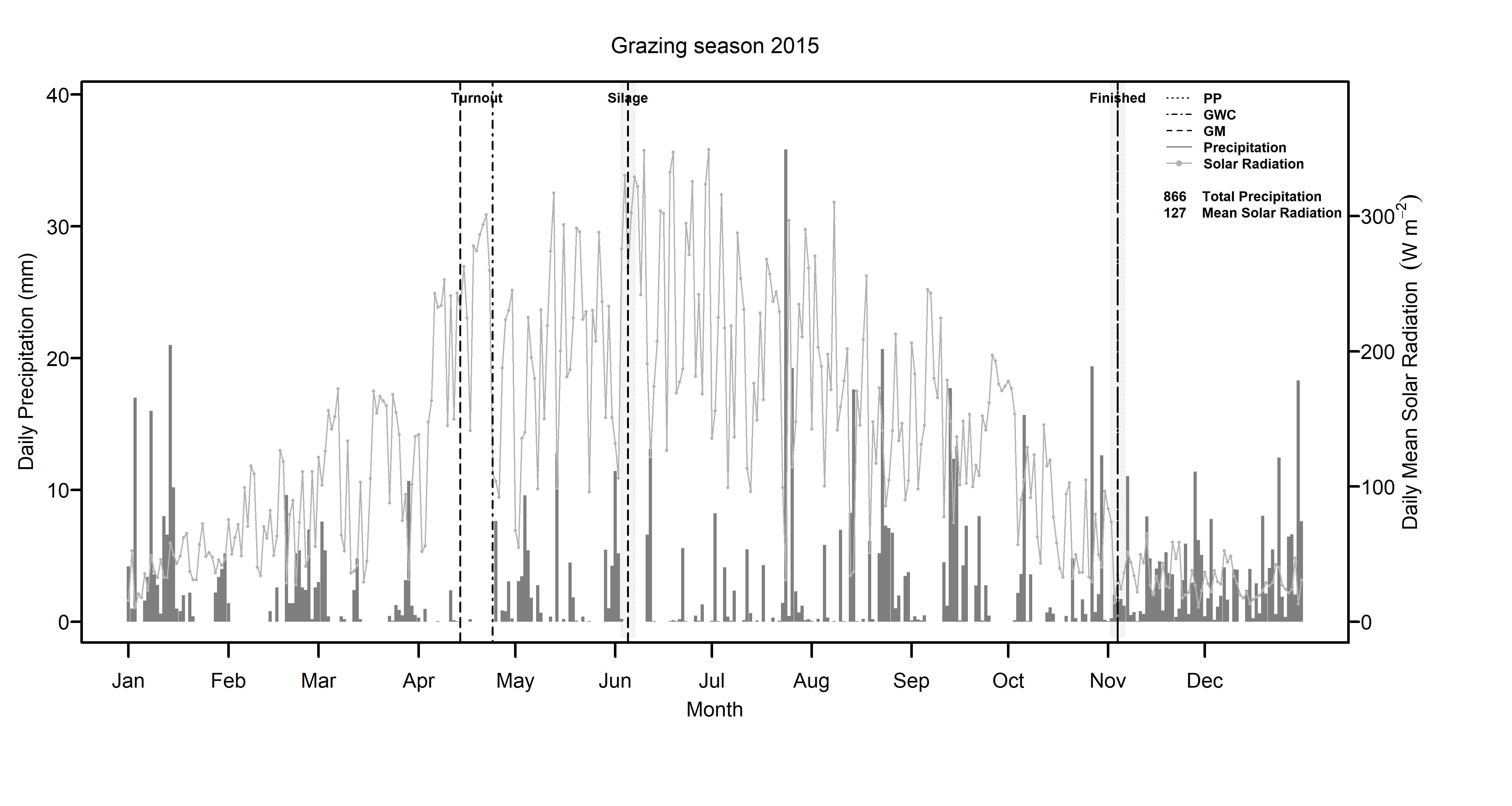


**
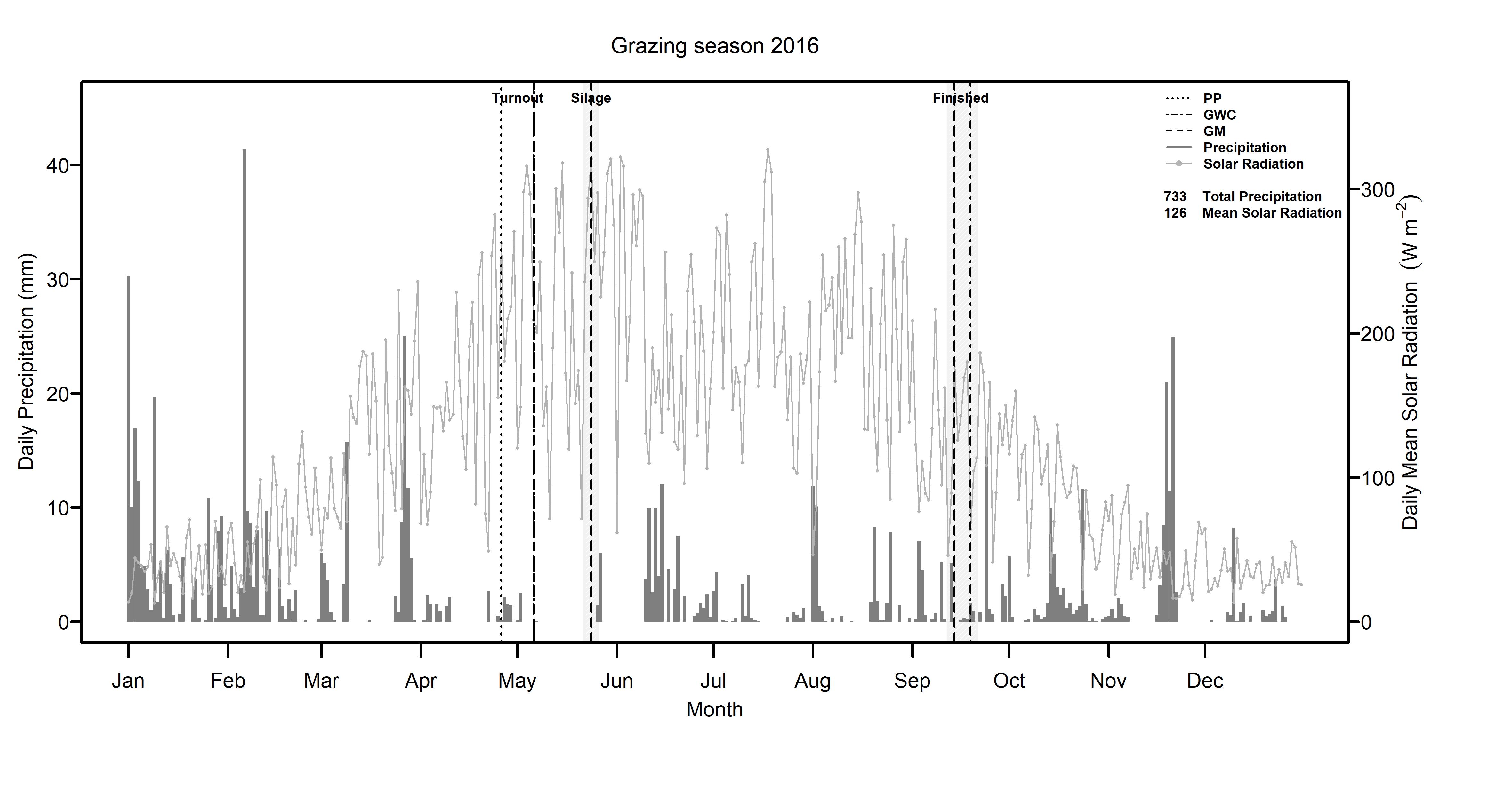
**

**
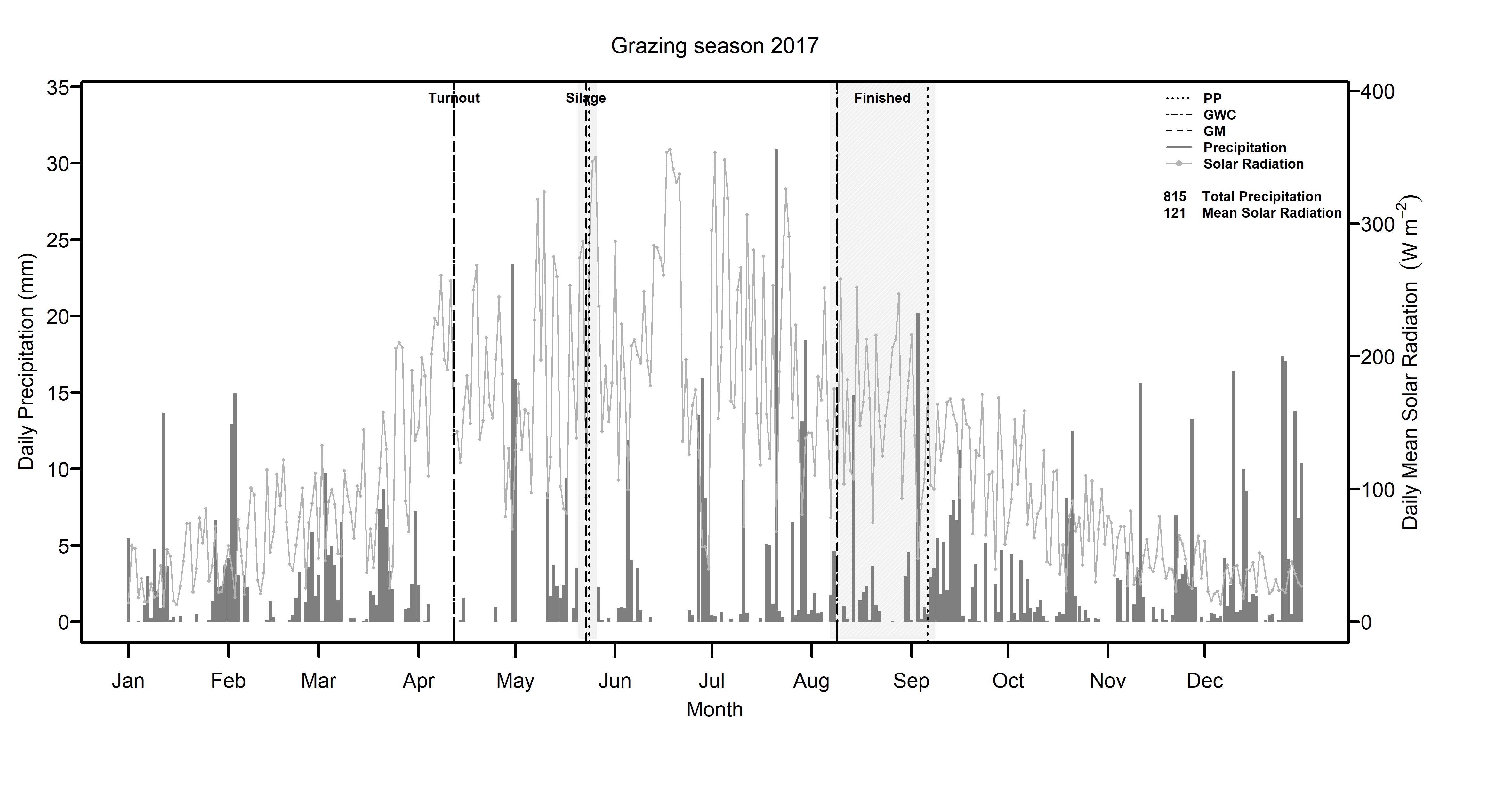
**

**Supplementary Table S1**.

Fatty acid (FAs), vitamin E and mineral composition of the silage offered (winter) to and the pasture grazed (spring-summer) by cattle, averaged across the three pasture-based systems for each of the three years.

|  | Silage | | | Pasture | | |
| --- | --- | --- | --- | --- | --- | --- |
|  |  | | |  | | |
|  | 2015 | 2016 | 2017 | 2015 | 2016 | 2017 |
| Fatty acids (g/kg DM) |  |  |  |  |  |  |
| C12:0 | 0.082 | 0.100 | 0.080 | 0.105 | 0.123 | 0.102 |
| C14:0 | 0.165 | 0.203 | 0.183 | 0.153 | 0.180 | 0.145 |
| C16:0 | 3.21 | 3.61 | 3.65 | 4.84 | 4.79 | 4.79 |
| C16:1 n-7 | 0.175 | 0.222 | 0.224 | 0.057 | 0.057 | 0.059 |
| C18:0 | 0.344 | 0.367 | 0.380 | 0.555 | 0.508 | 0.518 |
| C18:1 n-9 | 0.600 | 0.553 | 0.648 | 0.658 | 0.625 | 0.651 |
| C18:1 n-7 | 0.135 | 0.145 | 0.152 | 0.108 | 0.111 | 0.109 |
| C18:2 n-6 | 2.91 | 3.21 | 3.31 | 3.30 | 3.38 | 3.35 |
| C18:3 n-3 | 8.59 | 10.1 | 9.87 | 16.0 | 17.6 | 16.1 |
| C20:0 | 0.147 | 0.148 | 0.157 | 0.172 | 0.167 | 0.169 |
| Sum FAs | 19.5 | 22.6 | 22.4 | 31.2 | 33.0 | 31.4 |
| Vitamin (mg/kg DM) |  |  |  |  |  |  |
| E | 76.2 | 71.3 | 86.7 | 40.4 | 38.1 | 41.1 |
| Macro minerals (g/kg DM) | |  |  |  |  |  |
| Ca | 7.69 | 7.12 | 7.00 | 7.81 | 5.75 | 4.72 |
| K | 24.3 | 25.0 | 26.7 | 29.2 | 28.3 | 29.8 |
| Mg | 1.62 | 1.87 | 1.57 | 1.75 | 1.73 | 1.40 |
| Na | 1.79 | 2.95 | 1.82 | 0.72 | 1.70 | 0.80 |
| P | 3.05 | 3.20 | 3.12 | 3.64 | 3.76 | 3.54 |
| S | 2.04 | 2.24 | 2.25 | 2.88 | 2.93 | 2.89 |
| Micro minerals (mg/kg DM) | | | | | |  |
| As | 0.239 | 0.251 | 0.253 | 0.153 | 0.096 | 0.164 |
| Co | 0.654 | 0.824 | 0.750 | 0.173 | 0.058 | 0.135 |
| Cr | 1.89 | 1.87 | 2.77 | 1.26 | 0.915 | 1.41 |
| Cu | 20.6 | 24.7 | 22.2 | 7.69 | 8.41 | 7.13 |
| Fe | 472 | 487 | 475 | 311 | 154 | 271 |
| Mo | 1.1 | 1.36 | 0.956 | 1.38 | 2.00 | 1.27 |
| Mn | 156 | 182 | 140 | 104 | 120 | 106 |
| Ni | 1.44 | 1.35 | 1.79 | 1.48 | 1.19 | 2.03 |
| Se | 0.231 | 0.247 | 0.243 | 0.063 | 0.069 | 0.071 |
| Zn | 51.8 | 70.6 | 59.4 | 23.8 | 27.4 | 24.0 |
| Non-nutritional elements (mg/kg DM) | | | | | |  |
| Al | 550 | 544 | 543 | 406 | 170 | 382 |
| Cd | 0.039 | 0.036 | 0.042 | 0.037 | 0.024 | 0.034 |
| Pb | 0.356 | 0.345 | 0.316 | 0.341 | 0.161 | 0.282 |
| Ti | 10.4 | 11.0 | 10.9 | 9.15 | 6.61 | 8.33 |

**Supplementary Table S2**.

*Fatty acids (FAs) percentage of the Longissimus dorsi of the cattle from the three pasture-based systems over three years.*

|  | PP | GWC | MG | SED | *P* (F_2,4_) |
| --- | --- | --- | --- | --- | --- |
| Fatty acid (%) | |  |  |  |  |
| C12:0 | 0.059 | 0.061 | 0.059 | 0.0043 | 0.860 |
| C14:0 | 2.21 | 2.32 | 2.24 | 0.122 | 0.645 |
| C15:0 | 0.534 | 0.500 | 0.588 | 0.0495 | 0.305 |
| C16:0 | 23.7 | 24.3 | 23.9 | 0.50 | 0.563 |
| C16:1 n-7 | 3.28 | 3.40 | 3.23 | 0.165 | 0.637 |
| C17:0 | 1.11 | 1.11 | 1.18 | 0.036 | 0.243 |
| C18:0 | 14.3 | 13.5 | 14.6 | 0.87 | 0.506 |
| C18:1 n-9 | 35.7 | 36.5 | 35.6 | 1.21 | 0.725 |
| C18:1 n-7 | 0.858 | 0.926 | 0.814 | 0.0383 | 0.100 |
| C18:1 trans 6+7+8+9 | 0.270 | 0.254 | 0.260 | 0.0137 | 0.532 |
| C18:1 trans 10+11 | 1.98 | 1.53 | 1.73 | 0.218 | 0.239 |
| C18:2 CLA | 0.517 | 0.457 | 0.436 | 0.0491 | 0.337 |
| C18:2 n-6 | 2.01 | 2.11 | 1.97 | 0.161 | 0.697 |
| C18:3 n-3 | 1.17 | 1.13 | 1.05 | 0.067 | 0.305 |
| C20:1 n-9 | 0.122 | 0.128 | 0.119 | 0.0117 | 0.753 |
| C20:3 n-6 | 0.209 | 0.226 | 0.203 | 0.0248 | 0.663 |
| C20:4 n-3 | 0.174 | 0.169 | 0.162 | 0.0135 | 0.722 |
| C20:4 n-6 | 0.792 | 0.789 | 0.826 | 0.0971 | 0.918 |
| C20:5 n-3 | 0.658 | 0.576 | 0.667 | 0.0517 | 0.266 |
| C22:4 n-6 | 0.053 | 0.058 | 0.052 | 0.0062 | 0.599 |
| C22:5 n-3 | 0.967 | 0.934 | 0.947 | 0.0709 | 0.901 |
| C22:6 n-3 | 0.135 | 0.116 | 0.135 | 0.0191 | 0.551 |
| Sum identified FAs | 93.4 | 93.7 | 93.4 | 0.28 | 0.527 |
| Sum SFAs | 40.3 | 40.1 | 40.7 | 0.61 | 0.615 |
| Sum MUFAs | 42.2 | 42.7 | 41.7 | 1.10 | 0.688 |
| Sum n-3 PUFAs | 3.11 | 2.92 | 2.96 | 0.191 | 0.634 |
| Sum n-6 PUFAs | 3.06 | 3.19 | 3.05 | 0.284 | 0.874 |
| Sum PUFAs | 6.17 | 6.11 | 6.02 | 0.457 | 0.946 |
| Ratios |  |  |  |  |  |
| n-6:n-3 | 0.991 | 1.09 | 1.04 | 0.0484 | 0.264 |
| PUFA:SFA | 0.080 | 0.082 | 0.075 | 0.0051 | 0.446 |

Abbreviations: PP = permanent pasture; GWC = grass and white clover; MG = short-term monoculture grass ley; CLA = conjugated linoleic acid; SFAs = saturated fatty acids; MUFAs = monounsaturated fatty acids; PUFAs = polyunsaturated fatty acids.
